# Association between occupational hearing loss and predicted 10 year cardiovascular risk among middle aged industrial workers

**DOI:** 10.1371/journal.pone.0354726

**Published:** 2026-07-29

**Authors:** Negin Kassiri, Mohammad Eslami, Yasser Labbafinejad, Motahareh Harati, Saber Mohammadi, Zahra Asadgol

**Affiliations:** 1 Occupational Medicine Research Center, Iran University of Medical Sciences, Tehran, Iran; 2 Department of Occupational Medicine, School of Medicine, Iran University of Medical Sciences, Tehran, Iran; 3 Health Deputy, Iran University of Medical Sciences, Tehran, Iran; School of Pharmacy, Ardabil University of Medical Sciences, IRAN, ISLAMIC REPUBLIC OF

## Abstract

**Purpose:**

Occupational hearing loss is a prevalent occupational disease with potential systemic health implications, including cardiovascular disease (CVD). This cross-sectional study investigated the association between hearing loss and 10-year CVD risk among middle-aged industrial workers in Tehran.

**Methods:**

A total of 988 workers (83.7% male, mean age 46.8 ± 5.9 years) underwent routine occupational health examinations, including pure-tone audiometry and cardiovascular risk assessment using the WHO/ISH prediction chart. Hearing-loss phenotypes included low-frequency hearing loss (LFHL), high-frequency hearing loss (HFHL), overall hearing loss (HL), and noise-induced hearing loss (NIHL). Ear-specific hearing loss was first assessed separately for the left and right ears; participant-level hearing-loss variables were then defined based on the presence of hearing loss in either ear. The WHO/ISH 10-year CVD risk score was reported descriptively. To avoid conceptual overlap between the composite WHO/ISH score and its component variables, the composite CVD-risk variable was not entered into multivariable logistic regression models. Instead, multivariable logistic regression was used to identify demographic, occupational, and clinical factors associated with each hearing-loss phenotype.

**Results:**

The prevalence of NIHL was 19.9%. Ear-specific HFHL was observed in 27.2% of left ears and 25.5% of right ears, while LFHL was observed in 17.0% of left ears and 18.0% of right ears. In multivariable logistic regression models, sex and age were the most consistent independent factors associated with hearing loss. Female sex was associated with lower odds of LFHL, HFHL, overall HL, and NIHL compared with male sex. Older age was independently associated with LFHL, HFHL, and overall HL. Smoking was independently associated with HFHL and NIHL, while work experience was independently associated with NIHL. Job type and diabetes were not consistent independent predictors in the adjusted models. The composite WHO/ISH CVD-risk score was not included in the adjusted models to avoid conceptual overlap with its component variables.

**Conclusion:**

These findings highlight the importance of demographic, behavioral, and occupational factors, particularly sex, age, smoking, and work experience, in occupational hearing loss among middle-aged industrial workers. Although predicted 10-year CVD risk was described in this occupational cohort, the composite WHO/ISH risk score was not included in multivariable models together with its component variables. Hearing conservation programs should integrate noise control, regular audiometric screening, smoking cessation, and cardiovascular risk assessment as complementary components of occupational health surveillance.

## 1. Introduction

Occupational noise exposure is a major public health concern, as noise-induced hearing loss (NIHL) is one of the most common occupational diseases, affecting millions of workers across various industries, particularly in manufacturing, construction, mining, and transportation sectors [1]. It is estimated that approximately 16% of adult-onset hearing loss globally is attributable to occupational noise exposure [2,3]. The World Health Organization (WHO) has warned that more than 700 million people will experience disabling hearing loss by 2050, underscoring the growing burden of NIHL in both developed and developing countries [4]. The prevalence of NIHL varies widely depending on industry type, exposure intensity, and the effectiveness of preventive regulations, ranging from 10% to over 60% among occupational cohorts [5,6]. In low- and middle-income countries, particularly in parts of Asia, the prevalence is often higher due to inadequate enforcement of occupational safety standards and limited access to protective equipment [7].

Beyond its direct auditory consequences, occupational noise exposure imposes significant economic and psychosocial burdens by reducing productivity, increasing absenteeism, and contributing to communication difficulties, social isolation, and depressive symptoms [1,8]. Moreover, chronic noise exposure has been shown to activate stress responses, disturb autonomic nervous system balance, increase oxidative stress, and impair endothelial function [9,10,11]. Accumulating evidence suggests that the consequences of occupational noise exposure extend beyond hearing impairment to systemic health outcomes. Several studies have demonstrated that industrial workers exposed to high noise levels (>85 dB[A]) show a higher prevalence of both hearing impairment and hypertension, with exposure duration longer than 10 years further amplifying these risks [12]. Furthermore, a systematic review and meta-analysis of high-quality prospective studies revealed that occupational noise exposure significantly increases the risk of developing hypertension (HR ≈ 1.68) and cardiovascular disease (RR ≈ 1.34) compared to lower or non-exposed workers [13].

Cardiovascular Diseases (CVDs) are the leading cause of global mortality, responsible for more than 17 million deaths annually, accounting for nearly 30% of all deaths worldwide [14,15]. The burden is particularly pronounced in low- and middle-income countries, where premature deaths during the most productive years of life generate profound socioeconomic impacts [14]. Epidemiological studies have reported associations between chronic noise exposure or NIHL and elevated risks of hypertension, dyslipidemia, diabetes, and ischemic heart disease [16–19]. Several biological mechanisms have been proposed, including activation of the hypothalamic–pituitary–adrenal axis, increased sympathetic nervous system activity, oxidative stress, endothelial dysfunction, and disruption of cochlear microcirculation, all of which contribute to metabolic dysregulation and vascular injury [19–21].

Nonetheless, findings across studies remain inconsistent. Some investigations confirm strong associations between NIHL and adverse cardiovascular outcomes, whereas others fail to establish significant links [6,22,23]. This inconsistency may arise from differences in study populations, exposure assessment methods, or confounding variables such as smoking, age, or occupational category [19]. Importantly, while some evidence on the association between occupational noise exposure and cardiovascular health exists, research from middle-income regions—particularly the Middle East—remains very limited [24,25,26]. Despite extensive preventive strategies such as engineering controls, periodic noise monitoring, and hearing protection devices, NIHL remains largely irreversible, highlighting the urgent need for primary prevention in occupational health systems [27,28]. Therefore, further investigation of the relationship between occupational hearing loss and cardiovascular health among middle-aged industrial workers in Iran is essential. Such evidence could inform occupational health policies, support the development of integrated preventive strategies, and guide the revision of existing hearing conservation programs to address broader cardiovascular risks.

## 2. Methods

### 2.1. Study design and population

This study employed a cross-sectional design to investigate the association between occupational hearing loss and cardiovascular health among middle-aged employees. Participants were recruited from workers undergoing their routine annual occupational health examinations at an occupational medicine center located in the Azadi Industrial Zone. All individuals attending annual health assessments and referred this center between October 1, 2024 and March 30, 2025 were considered eligible for participation. In this study, a convenience sampling approach was used, and a total of 988 workers interred to the study. Before inclusion, all potential participants were briefed about the study objectives, procedures, confidentiality safeguards, and the voluntary nature of participation. Written informed consent was obtained from each participant, ensuring that they fully understood that their decision to participate—or not—would not affect their employment status or access to occupational health services. Inclusion criteria consisted of: (i) being currently employed at the time of the study, (ii) having at least one year of job tenure, and (iii) being over 40 years of age. To ensure the internal validity of the cardiovascular risk assessment, individuals with a documented history of cardiovascular disease were excluded from the analysis, as the WHO/ISH risk prediction charts are intended for primary prevention. Additionally, to minimize confounding factors affecting hearing thresholds, the exclusion criteria included a history of using ototoxic medications (such as aminoglycosides or high-dose loop diuretics) and any significant non-occupational ear pathology as reported in their medical records. Also, the other exclusion criterion was unwillingness to participate. All data used in the study were extracted from routine occupational health records, with no additional financial or clinical burden imposed on the workers. As part of these routine occupational health evaluations, workplace noise exposure had been previously assessed and documented by certified occupational hygienists through standardized walk‑through surveys and environmental noise measurements. These records confirmed the presence of hazardous noise levels in the work environment, although individual-level quantitative exposure metrics such as personal noise dosimetry were not available.

### 2.2. Data collection procedures

Data were obtained from the occupational health record, complemented by audiometric and laboratory assessments.

#### Demographic and occupational data.

Age, sex, job title, job tenure, smoking history, history of diabetes, hypertension, and body mass index (BMI) were extracted from health records. Smoking intensity was calculated in pack-years.

#### Occupational noise exposure documentation.

Information regarding exposure to hazardous workplace noise was extracted from occupational health records, where such exposure had been confirmed through periodic assessments conducted by certified occupational hygienists. Hazardous noise exposure was defined according to national occupational safety standards as exposure to sound levels ≥85 dB(A) over an 8-hour time-weighted average. However, individual-level data on hearing protection device use, leisure-time noise exposure, ototoxic medications, and potential chemical co-exposures were not available in the dataset.

#### Hearing assessment.

Hearing thresholds were evaluated by trained audiologists using pure-tone audiometry in a soundproof booth that met ambient noise standards for audiometric testing. Air-conduction thresholds were measured bilaterally at 500, 1000, 2000, 3000, 4000, 6000, and 8000 Hz using a calibrated clinical audiometer (Model: ASA 400 Advance Screening Audiometer (Iran)) using TDH‑39 supra-aural earphones. The audiometric equipment was calibrated periodically in accordance with international standards (ISO 389 for reference equivalent threshold sound pressure levels and ISO 8253−1 for pure-tone audiometry procedures). Daily biological calibration checks were also performed to ensure measurement consistency. All assessments were conducted following at least 12 hours without occupational noise exposure to avoid temporary threshold shifts. Thresholds were determined using the standard ascending method, defined as the lowest intensity level at which the participant responded to at least 50% of tone presentations. Mean thresholds were calculated separately for low frequencies (500–3000 Hz) and high frequencies (4000–8000 Hz). Hearing loss was defined as a mean threshold >25 dB in the corresponding frequency range. Noise-induced hearing loss (NIHL) was defined as the presence of a notch at 4000 or 6000 Hz with recovery at higher frequencies. In the present study, the term “occupational hearing loss” refers to hearing loss observed among workers employed in documented high-noise occupational environments, based on occupational health surveillance records. However, due to the absence of individual-level noise dosimetry data, this classification should be interpreted as reflecting hearing loss occurring in high-noise occupational settings rather than definitive individual causal attribution.

#### Cardiovascular risk assessment.

The 10-year risk of cardiovascular disease (CVD) was estimated using the WHO/ISH risk prediction chart for the Eastern Mediterranean Region B. Variables included in the algorithm were age, sex, systolic blood pressure, smoking status, diabetes status, and serum cholesterol levels.

### 2.3. Variables and measurements

The primary outcomes were four binary participant-level hearing-loss phenotypes derived from pure-tone audiometry: low-frequency hearing loss (LFHL), high-frequency hearing loss (HFHL), overall hearing loss (HL), and noise-induced hearing loss (NIHL). Ear-specific LFHL and HFHL were first calculated separately for the left and right ears. For participant-level analyses, LFHL was defined as the presence of LFHL in either ear, and HFHL was defined as the presence of HFHL in either ear. Overall HL was defined as the presence of either participant-level LFHL or participant-level HFHL. NIHL was defined according to the presence of a characteristic audiometric notch in at least one ear. Ear-specific findings were reported descriptively, whereas all regression analyses were performed using participant-level hearing-loss outcomes.

The 10-year risk of cardiovascular disease was estimated using the WHO/ISH risk prediction chart for the Eastern Mediterranean Region B. Variables included in the WHO/ISH algorithm were age, sex, systolic blood pressure, smoking status, diabetes status, and serum cholesterol level. For descriptive purposes, estimated CVD risk was reported in five categories: < 5%, 5–10%, 10–20%, 20–30%, and >30%. In descriptive comparisons, CVD risk was also categorized as <10% versus ≥10%. However, the composite WHO/ISH CVD-risk variable was not entered into multivariable regression models together with its component variables.

Covariates included demographic, occupational, and clinical factors, including age, sex, job type, work experience, smoking status, diabetes, hypertension, and BMI, depending on the specific model. Job type was categorized as white-collar versus blue-collar. Smoking status was categorized as current/former/never, and cumulative smoking exposure was recorded in pack-years for descriptive analyses. Clinical variables included diabetes and hypertension status.

### 2.4. Statistical analysis

Data were analyzed using SPSS version 24. Continuous variables were expressed as mean ± standard deviation, while categorical variables were reported as frequencies and percentages. Group differences were examined using independent t-tests for continuous variables and Chi-square tests for categorical variables.

To address potential conceptual overlap between the WHO/ISH composite CVD-risk score and its component variables, the composite WHO/ISH risk score was not included in the multivariable regression models. Instead, demographic, occupational, and clinical predictors were modeled individually. Multivariable logistic regression was used to identify factors independently associated with each participant-level hearing-loss phenotype. Separate models were constructed for LFHL, HFHL, overall HL, and NIHL. Candidate variables were selected based on clinical relevance, occupational plausibility, and prior evidence.

Multicollinearity among independent variables was assessed before interpretation of the final models. Model estimates were reported as regression coefficients, standard errors, Wald statistics, odds ratios, 95% confidence intervals, and p-values. A two-sided p-value <0.05 was considered statistically significant.

### 2.5. Ethical considerations

The study protocol was reviewed and approved by an accredited institutional ethics committee (Approval Code: IR.IUMS.REC.1403.527). All participants were informed about the study objectives, procedures, potential risks, and confidentiality safeguards prior to enrollment. Written informed consent was obtained from each participant, and participation was entirely voluntary. Individuals were assured that refusal to participate would not affect their employment status or access to occupational health services. All data were fully anonymized before analysis to ensure privacy and confidentiality. No additional financial costs or clinical procedures were imposed on participants, as all assessments were derived from routine occupational health examinations. The study involved only adult participants aged 18 years and older; no minors were included.

## 3. Results

### 3.1. Descriptive characteristics of participants

A total of 988 industrial workers were included in the study. The majority of participants were male (83.7%), with females representing 16.3% of the cohort. Regarding job type, 66.3% were employed in blue-collar positions, whereas 33.7% held white-collar roles. Approximately one-quarter of the participants (24.4%) reported a history of smoking. The mean age of participants was 46.8 ± 5.9 years (range: 40–76), with an average job experience of 9.2 ± 11.9 years (range: 1–53). The mean cumulative smoking exposure among smokers was 9.8 ± 10.0 pack-years (range: 1–50). The average body mass index (BMI) was 27.1 ± 4.0 kg/m², ranging from 16 to 52 kg/m². These descriptive findings are summarized in Table 1.

**Table 1:** Descriptive analysis of qualitative and quantitative variables.

Variables		Frequency (percentage)
**Sex**	Male	827 (83.7%)
	female	161 (16.3%)
**Job**	White collar	333 (33.7%)
	Blue collar	655 (66.3%)
**Smoking history**	Yes	241 (24.4%)
	No	747 (75.6%)
**Variables**	**Mean (±SD)**	**Min**-**Max**
**Age (year)**	46.75 (±5.9)	40-76
**Experience (year)**	9.24 (±11.9)	1-53
**Smoking (pack.year)**	9.80 (±10.0)	1-50
**BMI (kg/m**^**2**^)	27.07 (±4.0)	16-52

### 3.2. Ten-year cardiovascular disease risk distribution

**Fig 1:**
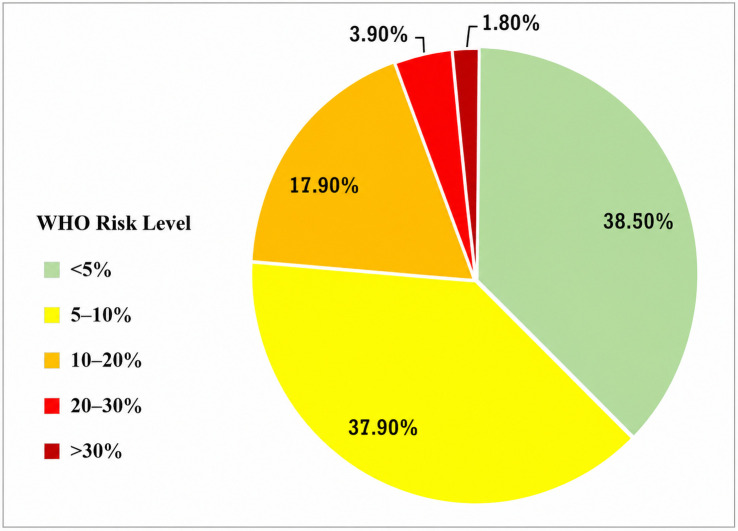
Ten-year risk of cardiovascular disease among the study population.

Based on the WHO/ISH risk classification, 38.5% of participants were categorized in the < 5% 10-year CVD risk group (n = 380), 37.9% in the 5–10% group (n = 374), 17.9% in the 10–20% group (n = 177), 3.9% in the 20–30% group (n = 39), and 1.8% in the > 30% group (n = 18). Overall, 76.4% of the study population had a 10-year CVD risk below 10% (n = 754), whereas 23.6% were classified as having a risk of 10% or higher (n = 234). The detailed percentage distribution of participants across risk categories is presented in Fig 1.

### 3.3. Hearing loss in the study population

**Fig 2:**
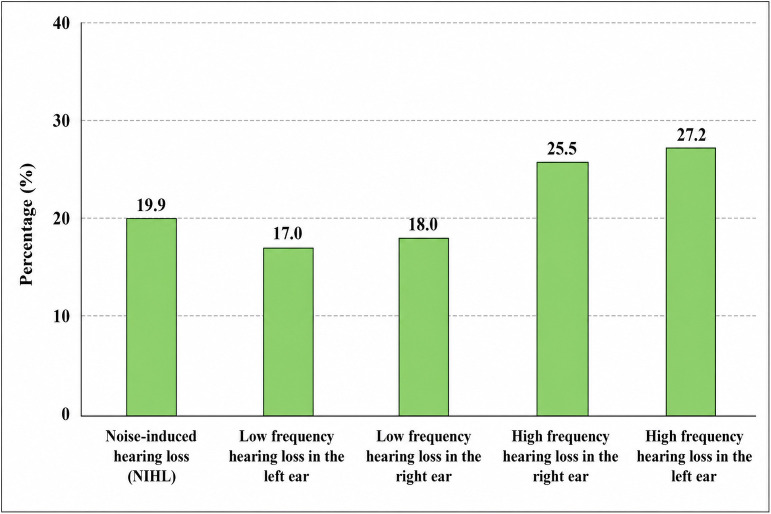
Percentage of types of hearing loss (NIHL, low frequency and high frequency) in the right and left ears in the study population.

Audiometric evaluation showed that 19.9% of participants had noise-induced hearing loss (NIHL), while 80.1% did not. Low-frequency hearing loss (LFHL) was observed in 17.0% of left ears and 18.0% of right ears. In contrast, high-frequency hearing loss (HFHL) was more prevalent, affecting 27.2% of left ears and 25.5% of right ears. Overall, high-frequency hearing loss was the most common type of hearing impairment in this cohort. The detailed percentage distribution of hearing loss types is also illustrated in Fig 2.

### 3.4. Association between demographic/clinical factors and hearing loss

Characteristics of participants according to hearing loss status are summarized in Table 2. Male sex was significantly associated with all hearing loss categories (LFHL, HFHL, HL, and NIHL; all p < 0.001). Blue-collar workers showed a higher prevalence of LFHL, HFHL, and overall hearing loss compared with white-collar workers (p = 0.003, p < 0.001, and p < 0.001, respectively), whereas no significant association was observed between job type and NIHL (p = 0.080). A history of smoking was significantly associated with all hearing loss outcomes, including LFHL (p = 0.009), HFHL (p < 0.001), HL (p < 0.001), and NIHL (p = 0.002). Diabetes was significantly associated with LFHL (p = 0.020) but not with HFHL, HL, or NIHL. Hypertension showed no significant association with any hearing loss category. Participants with a higher 10-year cardiovascular disease risk were more likely to exhibit HFHL, overall hearing loss, and NIHL (p < 0.001, p < 0.001, and p = 0.046, respectively), while the association between cardiovascular risk and LFHL did not reach statistical significance (p = 0.064).

**Table 2:** Characteristics of participants according to hearing loss status (LFHL, HFHL, HL, NIHL).

Variable		LFHL	HFHL	HL	NIHL
**Gender**	**Male**	217 (94.3%)	314 (93.7%)	347 (94.0%)	185 (93.9%)
	**Female**	13 (5.7%)	21 (6.3%)	22 (6.0%)	12 (6.1%)
	**p-value**	0.000	0.000	0.000	0.000
**Job**	**White collar**	59 (25.7%)	85 (25.4%)	93 (25.2%)	56 (28.4%)
	**Blue collar**	171 (74.3%)	250 (74.6%)	276 (74.8%)	141 (71.6%)
	**P value**	0.003	0.000	0.000	0.080
**Smoking history**	**Yes**	71 (30.9%)	109 (32.5%)	114 (30.9%)	65 (33.0%)
	**No**	159 (69.1%)	226 (67.5%)	255 (69.1%)	132 (67.0%)
	**P value**	0.009	0.000	0.000	0.002
**Diabetes**	**Yes**	23 (10.1%)	24 (7.3%)	29 (8.0%)	10 (5.2%)
	**No**	205 (89.9%)	305 (92.7%)	334 (92.0%)	182 (94.8%)
	**P value**	0.020	0.608	0.221	0.348
**Hypertension**	**Yes**	106 (46.1%)	162 (48.4%)	179 (48.5%)	85 (43.1%)
	**No**	124 (53.9%)	173 (51.6%)	190 (51.5%)	112 (56.9%)
	**P value**	0.638	0.101	0.066	0.616
**10-year CVD risk**	**Less than 10%**	186 (80.9%)	280 (83.6%)	306 (82.9%)	161 (81.7%)
	**More than 10%**	44 (19.1%)	55 (16.4%)	63 (17.1%)	36 (18.3%)
	**P value**	0.064	0.000	0.000	0.046

LFHL, HFHL, and overall HL were defined at the participant level based on the presence of hearing loss in either ear. P-values were calculated using Chi-square tests comparing participants with and without each hearing-loss phenotype.

### 3.5. Comparison of demographic and clinical variables between participants with and without hearing loss

Independent t-test analysis (Table 3) revealed significant differences in age and job experience between participants with and without hearing loss across all types. Specifically, participants with NIHL had a higher mean age (47.73 ± 6.09 years) compared to those without NIHL (46.51 ± 5.88 years, p = 0.010) and longer work experience (11.78 ± 13.68 vs. 8.61 ± 11.38 years, p = 0.001). Similarly, participants with LFHL, HFHL, or overall hearing loss (HL) were significantly older and had longer work experience than those without these conditions (all p < 0.001). Cumulative smoking exposure (pack-years) was significantly higher among participants with LFHL, HFHL, or HL compared to their counterparts (all p < 0.001), while the difference for NIHL did not reach statistical significance (p = 0.091). No significant differences were observed in BMI between participants with and without any type of hearing loss. Overall, these findings suggest that older age, longer job experience, and higher cumulative smoking exposure are associated with the presence of hearing impairment in this population, whereas BMI does not appear to be a major contributing factor (Table 3).

**Table 3:** Independent t-test results comparing mean demographic and clinical variables between participants with and without different types of hearing loss.

Variables	NIHL	Mean	Std. Deviation	P value
Age	No	46.51	5.881	0.010
	Yes	47.73	6.089	
Experience	No	8.61	11.379	0.001
	Yes	11.78	13.684	
Smoking (pack.year)	No	2.21	6.334	0.091
	Yes	3.09	7.119	
BMI	No	27.04	4.116	0.621
	Yes	27.20	4.006	
**Variables**	**LFHL**	**Mean**	**Std. Deviation**	**P value**
Age	No	46.03	5.366	0.000
	Yes	49.14	7.031	
Experience	No	8.49	11.158	0.000
	Yes	11.73	13.939	
Smoking (pack.year)	No	1.92	5.647	0.000
	Yes	3.92	8.592	
BMI	No	27.02	3.981	0.498
	Yes	27.23	4.448	
**Variables**	**HFHL**	**Mean**	**Std. Deviation**	**P value**
Age	No	45.46	4.834	0.000
	Yes	49.28	6.998	
Experience	No	7.70	10.461	0.000
	Yes	12.24	13.916	
Smoking (pack.year)	No	1.72	5.290	0.000
	Yes	3.70	8.231	
BMI	No	27.05	4.116	0.842
	Yes	27.11	4.053	
**Variables**	**HLtotal**	**Mean**	**Std. Deviation**	**P value**
Age	No	45.42	4.817	0.000
	Yes	48.99	6.907	
Experience	No	7.63	10.429	0.000
	Yes	11.95	13.698	
Smoking (pack.year)	No	1.66	5.017	0.000
	Yes	3.62	8.292	
BMI	No	26.98	3.949	0.356
	Yes	27.23	4.324	

### 3.6. Multivariable logistic regression analysis

Table 4 presents the results of multivariable logistic regression analyses examining demographic, occupational, and clinical factors associated with each participant-level hearing-loss phenotype. To avoid conceptual overlap, the composite WHO/ISH 10-year CVD-risk score was not included in the multivariable models together with its component variables.

**Table 4:** Multivariable logistic regression of demographic and clinical factors associated with different types of hearing loss (LFHL, HFHL, HL, and NIHL).

		B	S.E.	Wald	df	Sig.	Odds ratio	95% C.I. for OR	
								Lower	Upper
**LFHL**	**Sex**	−1.014	.314	10.451	1	.001	.363	.196	.671
	**Job**	−.205	.183	1.258	1	.262	.815	.569	1.166
	**Smoking**	.184	.177	1.082	1	.298	1.202	.850	1.702
	**Diabetes**	.381	.284	1.795	1	.180	1.463	.838	2.553
	**Age**	.069	.014	25.955	1	.000	1.072	1.044	1.101
	**Experience**	.001	.007	.015	1	.902	1.001	.988	1.014
	**Constant**	−3.145	.766	16.857	1	.000	.043		
**HFHL**	**Sex**	−.999	.258	14.954	1	.000	.368	.222	.611
	**Job**	−.237	.164	2.101	1	.147	.789	.572	1.087
	**Smoking**	.416	.165	6.363	1	.012	1.516	1.097	2.094
	**Age**	.094	.013	50.182	1	.000	1.099	1.070	1.128
	**Experience**	.009	.006	2.085	1	.149	1.009	.997	1.022
	**Constant**	−3.873	.717	29.167	1	.000	.021		
**HL**	**sex**	−1.168	.252	21.426	1	.000	.311	.190	.510
	**job**	−.294	.159	3.407	1	.065	.746	.546	1.018
	**smoking**	.279	.162	2.953	1	.086	1.322	.962	1.817
	**age**	.088	.013	45.176	1	.000	1.092	1.065	1.121
	**experience**	.008	.006	1.617	1	.203	1.008	.996	1.021
	**Constant**	−3.116	.701	19.743	1	.000	.044		
**NIHL**	**Sex**	−1.041	.320	10.552	1	.001	.353	.189	.662
	**Smoking**	.399	.180	4.911	1	.027	1.491	1.047	2.123
	**Age**	.011	.014	.575	1	.448	1.011	.983	1.040
	**Experience**	.015	.007	4.902	1	.027	1.015	1.002	1.029
	**Constant**	−1.005	.760	1.750	1	.186	.366		

Each hearing-loss phenotype was entered as a separate dependent variable. The composite WHO/ISH 10-year CVD-risk score was not included in these multivariable models to avoid conceptual overlap with its component variables. Sex was coded so that odds ratios below 1 indicate lower odds among females compared with males.

In the LFHL model, female sex was associated with lower odds of LFHL compared with male sex (OR=0.36, 95% CI: 0.20–0.67, p = 0.001), while older age was associated with higher odds of LFHL (OR=1.07, 95% CI: 1.04–1.10, p < 0.001). Job type, smoking status, diabetes, and work experience were not independently associated with LFHL.

In the HFHL model, female sex was associated with lower odds of HFHL compared with male sex (OR=0.37, 95% CI: 0.22–0.61, p < 0.001). Older age was independently associated with higher odds of HFHL (OR=1.10, 95% CI: 1.07–1.13, p < 0.001), and smoking was also independently associated with HFHL (OR=1.52, 95% CI: 1.10–2.09, p = 0.012). Job type and work experience were not independently associated with HFHL.

In the overall HL model, female sex was associated with lower odds of hearing loss compared with male sex (OR=0.31, 95% CI: 0.19–0.51, p < 0.001), and older age was associated with higher odds of overall HL (OR=1.09, 95% CI: 1.07–1.12, p < 0.001). Smoking showed a borderline association with overall HL (OR=1.32, 95% CI: 0.96–1.82, p = 0.086), whereas job type and work experience were not significant independent predictors.

In the NIHL model, female sex was associated with lower odds of NIHL compared with male sex (OR=0.35, 95% CI: 0.19–0.66, p = 0.001). Smoking was independently associated with NIHL (OR=1.49, 95% CI: 1.05–2.12, p = 0.027), and longer work experience was also associated with higher odds of NIHL (OR=1.02, 95% CI: 1.00–1.03, p = 0.027). Age was not independently associated with NIHL after adjustment.

In summary, the findings indicate that the prevalence of hearing loss, particularly high-frequency hearing loss (HFHL) and noise-induced hearing loss (NIHL), is significantly associated with demographic factors (such as male sex) and clinical factors (including age and 10-year CVD risk). These results not only confirm the role of age and sex in hearing impairment but also suggest a potential link between cardiovascular risk and auditory dysfunction in this industrial worker population, providing a foundation for clinical interpretation.

## 4. Discussion

This study aimed to examine the association between occupational noise-induced hearing loss (NIHL) and cardiovascular disease (CVD) risk among middle-aged industrial workers. Given the cross‑sectional design of the present study, the findings should be interpreted as associations rather than causal relationships between hearing loss and cardiovascular risk. Similar epidemiological studies have emphasized that cross‑sectional analyses cannot determine the temporal direction between exposure and outcome [29,30]. The findings indicated that older age and longer duration of occupational exposure were significantly associated with an increased likelihood of multiple forms of hearing loss, including low-frequency hearing loss (LFHL), high-frequency hearing loss (HFHL), overall hearing loss, and NIHL. In multivariable logistic regression models, each additional year of age was associated with a significantly higher odds ratio for these types of hearing impairment. Furthermore, high-frequency and overall hearing loss were significantly related to an elevated 10-year CVD risk, even after adjusting for demographic and clinical factors, while male sex also emerged as a consistent predictor of hearing loss in noise-exposed workers. Smoking exposure, expressed as pack-years, was associated with different types of hearing loss in descriptive and t-test analyses and was statistically significant in the adjusted model for LFHL (OR = 1.028, p = 0.049). In contrast, job category, diabetes, and hypertension did not show significant effects in the multivariable models.

The study population was predominantly composed of men (83.7%), reflecting the sex distribution typical of industrial and blue-collar occupations. Previous evidence has shown that men are at a higher risk of occupational noise-induced hearing loss (NIHL) due to their generally greater exposure to noisy environments [31,8]. This male predominance may have influenced the regression outcomes, where sex appeared as a protective factor for women (OR < 1). The mean age of participants was 46.7 years, with a relatively narrow range (40–76 years), indicating that most individuals were middle-aged and actively employed. Since age is an independent determinant of both age-related hearing loss (presbycusis) and cardiovascular diseases [32], this age distribution could act as a potential confounding factor in the observed associations. Although age was included as a continuous variable in all multivariable models, it may still reflect underlying biological aging processes that influence both auditory and cardiovascular systems [33]. In terms of job type, two-thirds of the study population were blue-collar workers. This finding is of particular occupational health relevance, as blue-collar workers are typically exposed to higher levels of industrial noise. Several studies [1] have demonstrated that industrial employees are at greater risk of NIHL and work‑related cardiovascular diseases compared with white-collar workers. Regarding smoking behavior, approximately one-quarter of participants reported a history of smoking, with a mean cumulative exposure of 9.8 pack-years. This represents a moderate level of tobacco exposure; however, previous research indicates that even exposures below 10 pack-years are associated with an increased risk of cardiovascular disease and impaired hearing capacity [34,35]. Therefore, the observed near-significant association between smoking and LFHL in the multivariable model was expected. The mean duration of job experience was 9.2 years, with a wide range (1–53 years), indicating that some participants had a prolonged history of occupational noise exposure. This wide variability underscores the importance of considering work experience as a potential determinant in hearing loss analyses. The average body mass index (BMI) of 27.1 kg/m² placed the study population in the overweight category. Overweight and obesity are well-established risk factors for cardiovascular disease [36]; thus, the high prevalence of excess weight in this cohort aligns with global evidence and may partially explain the observed burden of cardiovascular risk among these workers. Overall, the baseline characteristics of this population — including a higher mean age, predominance of men and blue-collar workers, and a high prevalence of overweight and smoking history — place them at increased risk for occupational hearing loss and cardiovascular diseases, providing an appropriate context to investigate the association between these two health domains.

The findings of this study also showed that, according to the WHO/ISH classification, approximately 76.4% of participants were in the < 10% risk group, while nearly one-fourth (23.6%) were categorized as having a higher-risk (≥10%) for cardiovascular diseases. This indicates that although the majority of participants fell within the low-risk category, a considerable proportion of the population remained at elevated cardiovascular risk, underscoring the need for targeted preventive interventions. This pattern is consistent with previous reports from the general Iranian population and other countries in the region, where studies have shown that about 20–30% of middle-aged adults have a ≥ 10‑year CVD risk of ≥10% [37,38]. Underlying factors such as the high prevalence of obesity, smoking, and hypertension may explain this distribution. From an occupational health perspective, the presence of this level of cardiovascular risk among the working population is particularly important, as concurrent exposure to occupational noise and job stress may exacerbate cardiovascular outcomes [19]. Environmental and occupational noise has been increasingly recognized as a potential contributor to cardiovascular stress responses through mechanisms involving autonomic activation, sleep disturbance, and vascular dysfunction [8,19].

The results showed that 19.9% of participants had noise-induced hearing loss (NIHL). High-frequency hearing loss (HFHL) was significantly more prevalent than low-frequency hearing loss, affecting 27.2% of left ears and 25.5% of right ears. This pattern is consistent with existing epidemiological evidence, which indicates that occupational noise exposure primarily affects high frequencies (3–6 kHz) [1,3]. This is primarily due to the vulnerability of the basal cochlear hair cells to high-energy sounds in the high-frequency range [33]. In contrast, low-frequency hearing loss was less common (17–18%), likely reflecting physiological differences in cochlear sensitivity. Similar studies on industrial workers in Italy and Finland have also reported higher prevalence of HFHL compared to LFHL [31]. These findings suggest that occupational hearing screenings should particularly focus on high frequencies to enable early identification of NIHL and prevent progression to broader hearing impairment. Although univariate analyses in this study showed significant associations between sex, job type, smoking history, and 10-year cardiovascular disease (CVD) risk with all types of hearing loss (LFHL, HFHL, overall HL, and NIHL), diabetes was associated only with LFHL, whereas hypertension did not show significant relationships with any type of hearing loss.

According to our results, men had higher odds of all types of hearing loss compared to women, with ORs ranging from approximately 3.0 to 3.7, indicating that men are nearly three to four times more likely to experience hearing impairment. This finding is consistent with international evidence; for instance, a study published reported that male sex is a significant predictor of high-frequency hearing loss among workers exposed to occupational noise [39]. Job type (blue-collar vs. white-collar) also showed a significant effect: blue-collar workers exhibited significantly higher odds of LFHL, HFHL, and overall HL. This reflects the impact of environmental and occupational exposures, particularly to noise, vibration, or other physical hazards. Studies assessing the risk of NIHL in industrial settings have similarly identified job type as an independent risk factor [39]. Smoking history was another important factor in this study; smokers exhibited a higher prevalence of all types of hearing loss, and univariate analyses showed significant differences. This is in line with systematic reviews and meta-analyses—for example, a recent meta-analysis found that current smoking is associated with nearly a two-fold increased risk of NIHL compared to non-smokers [40]. Additionally, Mohammadi et al. [41] reported that individuals actively or passively exposed to tobacco smoke in noisy environments have a higher risk of developing NIHL [41]. These findings suggest that smoking may have a synergistic effect with noise exposure on auditory damage. Potential mechanisms include impaired microvascular perfusion of the cochlea and increased oxidative stress in the inner ear sensory cells, although the impact may vary depending on smoking intensity, age of initiation, and other environmental conditions [41]. Regarding 10-year cardiovascular disease (CVD) risk, participants with a risk ≥10% had higher odds of HFHL, overall HL, and NIHL, with the differences in HFHL and overall HL being particularly significant. The present study described predicted 10-year CVD risk using the WHO/ISH chart. However, because the WHO/ISH score is derived from age, sex, smoking status, diabetes, systolic blood pressure, and cholesterol, the composite score was not entered into the multivariable hearing-loss models together with its component variables. This revision avoided conceptual overlap and allowed the interpretation of individual demographic and clinical predictors of hearing loss. Therefore, the present findings should not be interpreted as showing an independent adjusted association between the composite WHO/ISH CVD-risk score and hearing loss. Rather, they suggest that several individual factors relevant to both cardiovascular health and auditory function, particularly age, sex, and smoking, are associated with hearing-loss phenotypes. While some studies suggest that vascular and metabolic risk factors may influence cochlear microcirculation and auditory function, the evidence linking composite cardiovascular risk scores directly to hearing loss remains limited and largely observational [33,19]. This interpretation remains biologically plausible. This finding aligns with recent evidence indicating an overlap between vascular diseases and auditory system damage. Recent research indicates that CVD risk factors such as hypertension, obesity, diabetes, and microvascular dysfunction in cochlear vessels can reduce blood flow, increase oxidative stress, and ultimately damage cochlear hair cells. Several observational studies have reported associations between cardiovascular risk factors and hearing impairment, suggesting possible vascular contributions to cochlear dysfunction [42,43]. Similarly, advancing age reflects both cochlear degeneration and cumulative biological vulnerability [44]. However, because the study was cross-sectional and lacked individual-level noise dosimetry, these mechanisms should be interpreted as plausible explanatory pathways rather than direct causal evidence from the present data.

Furthermore, noise exposure is an independent risk factor for cardiovascular disease. Even after adjusting for other risk factors such as hypertension, diabetes, or obesity are controlled, chronic noise exposure can lead to increased cardiovascular mortality, higher risk of ischemic heart disease (IHD) and stroke, endothelial dysfunction, and systemic inflammation. The underlying mechanisms include neural stress, elevated cortisol and adrenaline, chronic inflammation, impaired nitric oxide synthesis, and disruption of circadian rhythms. Therefore, noise directly affects the cardiovascular system and can contribute to elevated blood pressure, heart failure, arrhythmias, and other cardiovascular disorders [45–47]. Consequently, the combination of environmental noise exposure and the presence of cardiovascular risk factors may have a synergistic effect on auditory function. For instance, individuals working in noisy occupational settings who also have hypertension or diabetes may be at higher risk of hearing loss compared to those without these conditions. This interaction suggests that cardiovascular health can modulate the susceptibility of the auditory system to noise-induced damage. Diabetes, however, was only significantly associated with low-frequency hearing loss (LFHL), which may be explained by its primary effect on small vessels and microvascular function, leading initially to impairments at lower frequencies. High-frequency hearing loss, typically resulting from greater damage to hair cells and neural pathways, may be less affected or affected later. Similar patterns have been reported in other studies [39]. Moreover, hypertension did not show a significant association in this study, which could be due to several factors: the sample size of the hypertensive group may have been insufficient, or adjusting for confounding variables such as age, smoking, and noise exposure may have attenuated its detectable effect in univariate analyses. In studies where blood pressure is included as a controlled factor in multivariable models, its effect is usually weaker unless the severity of hypertension and treatment adherence are precisely accounted for [48].

Based on the results of independent t-tests, participants with various types of hearing loss had higher mean ages. This finding is consistent with previous evidence showing that advancing age is one of the strongest risk factors for hearing loss, which is attributed to degenerative changes in cochlear hair cells and neural pathways that intensify over time [39]. Work experience was also significantly higher in participants with all types of hearing loss, particularly in groups with HFHL and NIHL, reflecting the cumulative effect of prolonged occupational noise exposure. Numerous studies have demonstrated that longer durations of employment in noisy environments are directly associated with the severity and prevalence of hearing loss [49]. In contrast, body mass index (BMI) did not differ significantly between participants with and without hearing loss. This aligns with some studies that found no direct association between obesity and hearing loss, although evidence in this area remains mixed. Some research suggests that obesity may indirectly contribute to hearing loss through its effect on cardiovascular disease, while a direct effect, especially in occupational populations, remains less established [48].

Multivariable logistic regression analysis showed that female sex was associated with lower odds of all hearing-loss phenotypes, which is consistent with the generally higher occupational noise exposure experienced by men in industrial settings. Age was independently associated with LFHL, HFHL, and overall HL, reflecting the contribution of age-related cochlear and neural degeneration. This relationship aligns with the well-established pattern of age-related hearing loss (presbycusis). Multiple studies have shown that, over time, cochlear hair cells and neural pathways undergo gradual degeneration, which is particularly pronounced at high frequencies [50]. However, age was not independently associated with NIHL after adjustment, whereas work experience remained associated with NIHL. This finding is occupationally meaningful, as NIHL is expected to reflect cumulative exposure to workplace noise more directly than age alone [44]. Variables such as work experience and diabetes did not show significant effects in most models, which may be due to limited statistical power in certain models, especially for subgroups with relatively small sample sizes, such as participants with high CVD risk. Additionally, unmeasured factors such as individual variability in occupational noise exposure intensity, use of hearing protection devices, or non‑occupational noise exposure may also contribute to residual confounding in observational studies of occupational hearing loss.

### 4.1. Limitations and strengths

This study has several limitations that should be considered when interpreting the results. First, its cross-sectional design does not allow for establishing a causal relationship between noise-induced hearing loss (NIHL) and cardiovascular risk. Second, behavioral factors such as smoking history and occupational exposure were self-reported, which may be subject to recall bias. Third, although occupational noise exposure was documented in workers’ occupational health records based on routine evaluations by occupational health professionals, individual-level quantitative exposure data, such as personal noise dosimetry, were not available for analysis. In addition, information on hearing protection device use, leisure-time noise exposure, and potential chemical co-exposures was unavailable. Furthermore, although age was included as a continuous variable in all multivariable models to partially account for age-related hearing loss, it was not possible to fully distinguish NIHL from presbycusis, particularly because these conditions may share similar high-frequency audiometric patterns. Another limitation of this study is the potential for a healthy worker effect. Because the study population consisted of actively employed industrial workers undergoing routine occupational health examinations, individuals with severe health conditions or established cardiovascular disease may have already left employment. This selection may lead to an underestimation of the association between hearing loss and cardiovascular risk and may limit the generalizability of the findings beyond actively employed worker populations.

Nevertheless, the study also has important strengths: a large sample of middle-aged workers was examined, hearing and cardiovascular risk assessments were conducted using standardized and validated tools, and various hearing loss phenotypes were analyzed, allowing a more precise evaluation of the association between NIHL and cardiovascular factors. Furthermore, occupational noise exposure had been routinely assessed and documented within the industrial health surveillance system, supporting the relevance of this population as a noise-exposed working cohort. These findings can inform preventive strategies and occupational health programs in industrial settings [51–53].

## 5. Conclusion

The findings of this study indicate that hearing loss, particularly high-frequency hearing loss, was common among middle-aged industrial workers. Female sex was associated with lower odds of LFHL, HFHL, overall HL, and NIHL compared with male sex. Older age was independently associated with LFHL, HFHL, and overall HL, while smoking was independently associated with HFHL and NIHL. Work experience was independently associated with NIHL, supporting the role of cumulative occupational exposure in this phenotype. After revision of the analytical strategy, the composite WHO/ISH 10-year CVD-risk score was not included in multivariable models together with its component variables.

Therefore, assessing hearing risk and implementing preventive strategies should consider this factor. Such measures may include reducing noise exposure, managing cardiovascular risk factors, and conducting regular hearing screenings for high-risk populations. Routine hearing assessments should especially target middle-aged or older workers exposed to occupational noise. In addition, preventive and control interventions—such as the use of hearing protection devices, minimizing noise exposure, and public health programs for smoking cessation—can play a key role in reducing the burden of hearing loss.
